# Supplementary material for: Factors Influencing the Intention to Sign Up for Advanced Care Planning in Day Care for Psychiatric Patients
Source: Nurs Rep. 2023 Jun 6;13(2):865–76. doi: 10.3390/nursrep13020076 (PMC10303737; doi:10.3390/nursrep13020076)
Supplement: Supplementary file 1 [file nursrep-13-00076-s001.zip › Supplementary File S1.pdf]

Supplementary documents

Survey Questionnaire

**Survey Questionnaire：** Number:□ □ □
Good morning/afternoon, sir/madam, this is Ms Yi-Chien Chen, an interviewer from Taipei Veterans General Hospital Yuli Branch, Hualien of Taiwan. The title of our stusy is " An Investigation of Correlated Factors for Intention to Sign Up for Advanced Care Planning among Psychiatric Patients in A Day Care Center". The aim of our study is to investigate the factors affecting the intention of day ward patients to sign up for hospice and palliative care by using the survey questionnaire with five major sections, (1) Demographic characteristics, (2) Hospice and Palliative Care Knowledge Scale, (3) Hospice and Palliative Care Attitude scale, (4) Experience of Hospice and Palliative Care , and (5) Hospice Palliative Care Intent to Sign Scale. For quality control purpose, our conversation may be recorded but will be destroyed shortly after our quality control process is complete.
Is it okay for us to start this survey? □Yes □No
Thank you for your participation.

1. Demographic characteristics

We would like to ask you about your personal information:
1. How old are you? ______years old
2. Gender? □Male □Female
3. Education Level? □Illiterate □Elementary School □Junior High School □Senior High School □Vocational School □University School □Graduate Schools and above
4. Religion? □No □Buddhism □Taoism □Christianity □Catholicism □Other:_________
5. Workplace Employment: □No □Yes
6. Physical Co-Morbidity: □No □Yes
7. Experience as a caregiver: □No □Yes

2.Hospice and Palliative Care Knowledge Scale

**There is no right or wrong answer for the following items to understand your knowledge of "hospice and palliative care", please read each item and check the one you think fits you the best.**
01 The Hospice Palliative Care Act has been enacted and its implementation has been announced in Taiwan.<br>□Yes □No □Unclear
02 The fundamental objective of hospice and palliative care is to actively enhance the quality of life of terminally ill patients and to provide hospice care.<br>□Yes □No □Unclear
03 Hospice and Palliative Care will respect the patient's wishes and communicate with the patient and family before performing any tests and treatments on the terminally ill.<br>□Yes □No □Unclear
04 The majority of pain and suffering experienced by dying patients can be significantly alleviated by hospice and palliative care.<br>□Yes □No □Unclear
05 Hospice and palliative care actively give the best suitable therapy to patients.<br>□Yes □No □Unclear
06 Hospice and palliative care also involve no withdrawal of medical treatment.<br>□Yes □No □Unclear
07 Hospice and palliative care are limited to the elderly and those with terminal conditions.

□Yes □No □Unclear

08 After a person has signed a permission form for hospice and palliative care, they cannot be withdrawn regardless of their condition.

□Yes □No □Unclear

09 Hospice and palliative care concentrate on the medical, psychological, and spiritual care of the entire patient throughout the last phase of an illness.

□Yes □No □Unclear

10 When a patient has a pre-established hospice and palliative care request, family members can make all medical choices and change the form's contents on the patient's behalf.

□Yes □No □Unclear

11 When adopting hospice and palliative care for terminally ill patients, physicians should tell patients or family members about their treatment goals.

□Yes □No □Unclear

12 Patients who have demonstrated a clear wish to be told about their condition should be informed with family members' permission.

□Yes □No □Unclear

13 The hospice and palliative care team is comprised of physicians, nurse practitioners, social workers, psychologists, and religious professionals.

□Yes □No □Unclear

3.Hospice and Palliative Care Attitude Scale

This section aims to understand your views and feelings about hospice and palliative care. There is no right or wrong answer, please mark ˇ in □ according to your level of agreement.

01 I think choosing hospice and palliative care will improve my quality of life at the end of my life rather than prolong it ineffectively.

(1)□Strongly disagree (2)□Disagree (3)□No opinion (4)□Agree (5)□Strongly agree

02 I think choosing hospice and palliative care can relieve my internal fears, anxiety and depressive reactions.

(1)□Strongly disagree (2)□Disagree (3)□No opinion (4)□Agree (5)□Strongly agree

03 I think if I choose hospice and palliative care, my life and future will be hopeless.

(1)□Strongly disagree (2)□Disagree (3)□No opinion (4)□Agree (5)□Strongly agree

04 How can the implementation of hospice and palliative care help me to communicate with my family and medical staff in advance about my medical decisions during terminal illness.

(1)□Strongly disagree (2)□Disagree (3)□No opinion (4)□Agree (5)□Strongly agree

05 I think if I choose hospice and palliative care, I feel abandoned by my family and medical staff.

(1)□Strongly disagree (2)□Disagree (3)□No opinion (4)□Agree (5)□Strongly agree

06 Hospice and palliative care reduces conflict of opinion between me and my family.

(1)□Strongly disagree (2)□Disagree (3)□No opinion (4)□Agree (5)□Strongly agree

07 I think the implementation of hospice and palliative care can reduce the meaningless waste of medical resources.

(1)□Strongly disagree (2)□Disagree (3)□No opinion (4)□Agree (5)□Strongly agree

08 Adults over 20 years of age sign a hospice and palliative care wish form for life with no expiration date.

(1)□Strongly disagree (2)□Disagree (3)□No opinion (4)□Agree (5)□Strongly agree

09 If a relative has recently given consent to replace a terminally ill patient who is in a coma of terminal consciousness, in terms of priority, parents come first before spouses.

(1)□Strongly disagree (2)□Disagree (3)□No opinion (4)□Agree (5)□Strongly agree

10 Medical staff provide terminal medical clinical care in accordance with my personal hospice and palliative care wishes.

(1)□Strongly disagree (2)□Disagree (3)□No opinion (4)□Agree (5)□Strongly agree

11 Receiving hospice and palliative care allow my wishes for terminal care to be respected.

(1)□Strongly disagree (2)□Disagree (3)□No opinion (4)□Agree (5)□Strongly agree

4.Experience of Hospice and Palliative Care

The following items are designed to understand your experience with Hospice and Palliative Care, and there are no right or wrong answers, please read through the descriptions and check your answers in the boxes

01 Hospitalization experience within the past five years.<br>□Yes □No<br>02 Hospitalization events of family members in the past five years.<br>□Yes □No<br>03 Any death of a family member in the past five years.<br>□Yes □No<br>04 Any death of a close friend in the past five years.<br>□Yes □No<br>05 Engaged in discussions regarding hospice and palliative care with others.<br>□Yes □No<br>06 Engaged in discussions regarding hospice and palliative care with medical professionals.<br>□Yes □No<br>07 Participated in "Hospice and Palliative Care" promotional activities.<br>□Yes □No<br>08 Heard about "hospice and palliative care".<br>□Yes □No<br>09 Registered wishes for hospice and palliative care on health insurance card.<br>□Yes □No

5.Hospice Palliative Care Intent to Sign Scale

**Upon completion of the above-mentioned information about "Hospice and Palliative Care", when the health care provider explains to you and asks you about your intention to sign the letter of intent, select the one that matches your personal preference and mark ˇ.**
01 The possibility that I will sign a "Do Not Resuscitate Letter of Intent".<br>(1) □Very unlikely (2) □Unlikely (3) □Uncertain (4) □Likely (5) □Very likely<br>02 The possibility that I will sign the "Hospice and Palliative Care" form.<br>(1) □Very unlikely (2) □Unlikely (3) □Uncertain (4) □Likely (5) □Very likely<br>03 The possibility that I will sign the "No CPR" form.<br>(1) □Very unlikely (2) □Unlikely (3) □Uncertain (4) □Likely (5) □Very likely<br>04 The possibility that I will sign the "No Life-Saving Medical Treatment".<br>(1) □Very unlikely (2) □Unlikely (3) □Uncertain (4) □Likely (5) □Very likely<br>05 The possibility that I will register my health insurance card for "hospice and palliative care".<br>(1) □Very unlikely (2) □Unlikely (3) □Uncertain (4) □Likely (5) □Very likely

Thank you for your help.

STROBE Statement—Checklist of items that should be included in reports of *cross-sectional studies*

	Item No	Recommendation	Page No
**Title and abstract**	1	(*a*) Indicate the study's design with a commonly used term in the title or the abstract	14-20
		(*b*) Provide in the abstract an informative and balanced summary of what was done and what was found	20-25
**Introduction**			
Background/rationale	2	Explain the scientific background and rationale for the investigation being reported	35-98
Objectives	3	State specific objectives, including any prespecified hypotheses	141-148
**Methods**			
Study design	4	Present key elements of study design early in the paper	101-114
Setting	5	Describe the setting, locations, and relevant dates, including periods of recruitment, exposure, follow-up, and data collection	116-120
Participants	6	(*a*) Give the eligibility criteria, and the sources and methods of selection of participants	120-134
Variables	7	Clearly define all outcomes, exposures, predictors, potential confounders, and effect modifiers. Give diagnostic criteria, if applicable	NA
Data sources/measurement	8*	For each variable of interest, give sources of data and details of methods of assessment (measurement). Describe comparability of assessment methods if there is more than one group	157-200
Bias	9	Describe any efforts to address potential sources of bias	131-134
Study size	10	Explain how the study size was arrived at	135-140
Quantitative variables	11	Explain how quantitative variables were handled in the analyses. If applicable, describe which groupings were chosen and why	162-200
Statistical methods	12	(*a*) Describe all statistical methods, including those used to control for confounding	202-211
		(*b*) Describe any methods used to examine subgroups and interactions	202-211
		(*c*) Explain how missing data were addressed	216-217
		(*d*) If applicable, describe analytical methods taking account of sampling strategy	203-206
		(*e*) Describe any sensitivity analyses	209-211

**Results**			
Participants	13*	(a) Report numbers of individuals at each stage of study—eg numbers potentially eligible, examined for eligibility, confirmed eligible, included in the study, completing follow-up, and analysed	216-217
		(b) Give reasons for non-participation at each stage	NA
		(c) Consider use of a flow diagram	154-155
Descriptive data	14*	(a) Give characteristics of study participants (eg demographic, clinical, social) and information on exposures and potential confounders	216-228
		(b) Indicate number of participants with missing data for each variable of interest	216-217
Outcome data	15*	Report numbers of outcome events or summary measures	213-259
Main results	16	(*a*) Give unadjusted estimates and, if applicable, confounder-adjusted estimates and their precision (eg, 95% confidence interval). Make clear which confounders were adjusted for and why they were included	260-278
		(*b*) Report category boundaries when continuous variables were categorized	NA
		(*c*) If relevant, consider translating estimates of relative risk into absolute risk for a meaningful time period	NA
Other analyses	17	Report other analyses done—eg analyses of subgroups and interactions, and sensitivity analyses	260-278
**Discussion**			
Key results	18	Summarise key results with reference to study objectives	360-363
Limitations	19	Discuss limitations of the study, taking into account sources of potential bias or imprecision. Discuss both direction and magnitude of any potential bias	393-404
Interpretation	20	Give a cautious overall interpretation of results considering objectives, limitations, multiplicity of analyses, results from similar studies, and other relevant evidence	325-350
Generalisability	21	Discuss the generalisability (external validity) of the study results	377-391
**Other information**			
Funding	22	Give the source of funding and the role of the funders for the present study and, if applicable, for the original study on which the present article is based	NA
